# A Systematic Review of the Psychosocial Adjustment of Children and Adolescents with Facial Palsy: The Impact of Moebius Syndrome

**DOI:** 10.3390/ijerph17155528

**Published:** 2020-07-30

**Authors:** Matthew Hotton, Esme Huggons, Claire Hamlet, Kathleen Bogart, David Johnson, Jonathan H. Norris, Sarah Kilcoyne, Louise Dalton

**Affiliations:** 1 Oxford Facial Palsy Service, Oxford University Hospitals NHS Foundation Trust, Oxford OX3 9DU, UK; eh675@bath.ac.uk (E.H.); david.johnson@ouh.nhs.uk (D.J.); sarah.kilcoyne@ouh.nhs.uk (S.K.); louise.dalton@psych.ox.ac.uk (L.D.); 2 Centre for Appearance Research, University of the West of England, Bristol BS16 1QY, UK; claire.hamlet@uwe.ac.uk; 3 School of Psychological Science, Oregon State University, Corvallis, OR 97331, USA; kathleen.bogart@oregonstate.edu; 4 Oxford Eye Hospital, Oxford University Hospitals NHS Foundation Trust, Oxford OX3 9DU, UK; jonathan.norris@ouh.nhs.uk

**Keywords:** facial palsy, psychosocial adjustment, Moebius syndrome

## Abstract

Introduction: Facial palsy is often associated with impaired facial function and altered appearance. However, the literature with regards to the psychological adjustment of children and adolescents with facial palsy has not been systematically reviewed to date. This paper aimed to review all published research with regards to psychosocial adjustment for children and adolescents with facial palsy. Methods: MEDLINE, CINAHL, Embase, PsychInfo and AMED databases were searched and data was extracted with regards to participant characteristics, study methodology, outcome measures used, psychosocial adjustment and study quality. Results: Five studies were eligible for inclusion, all of which investigated psychosocial adjustment in participants with Moebius syndrome, a form of congenital facial palsy. Many parents reported their children to have greater social difficulties than general population norms, with difficulties potentially increasing with age. Other areas of psychosocial adjustment, including behaviour, anxiety and depression, were found to be more comparable to the general population. Discussion: Children and adolescents with Moebius syndrome may experience social difficulties. However, they also demonstrate areas of resilience. Further research including individuals with facial palsy of other aetiologies is required in order to determine the psychosocial adjustment of children and adolescents with facial palsy.

## 1. Introduction

Facial palsy (FP) is characterised by damage to the facial nerve, resulting in weakness of the facial muscles. FP may be associated with a variety of acquired conditions including, but not limited to, Bell’s palsy, trauma, herpes zoster virus or acute otitis media [1]. In neonates, FP occurs in 0.23–1.8% of live births, of which 78–90% are associated with birth trauma [2]. There are also several congenital conditions associated with facial palsy, such as CHARGE syndrome and Moebius syndrome, the latter of which can also be associated with palsy of the sixth cranial nerve (resulting in impairment of eye movement), although other cranial nerves may also be affected [3]. The physical sequelae of FP include difficulties with eye closure, potentially resulting in corneal exposure and leading to blindness; difficulties eating and drinking; hearing and speech problems and pain [1]. The incidence of FP in the entire paediatric population is believed to be 21.1 per 100,000 [4].

Whether genetic in origin, congenital or acquired, impairments in facial function associated with FP can include difficulties producing effective facial expression of emotion [3]. Typical facial expression of emotion throughout the lifespan conveys important emotional information and is therefore central to effective social interactions [5]. Furthermore, facial mimicry and effective responses to infant facial emotional cues play important roles in the development of secure attachment relationships and effective communication [6]. Impairments in facial expression of emotion can be misinterpreted by others as boredom, a lack of confidence or disinterest [7]. This misinterpretation of the emotional state can subsequently result in fewer positive or effective social interactions [8].

In addition to the communication difficulties associated with impaired facial expression, the stigma experienced by children and young people with visible differences in facial appearance has been well documented [9], with associated discrimination potentially resulting in fear of negative evaluation by others and adverse effects on quality of life, self-esteem and body image [10]. For example, a focus group of adolescents with Moebius syndrome reported difficulties with social engagement, prejudice, low confidence and bullying [11].

A review by Hotton et al. [12] highlighted that adults with FP are more likely than the general population to experience psychological distress and poorer quality of life, especially in relation to social function. Given these findings, it is important to determine the extent of the psychosocial impact of FP within paediatric populations.

### Aims and Objectives

This paper aimed to review the literature regarding the psychosocial adjustment of children and adolescents with FP and identify factors associated with poorer psychosocial adjustment.

## 2. Methods

### 2.1. Protocol and Registration

Methods of data extraction and eligibility criteria were specified in advance of the review being.

### 2.2. Eligibility Criteria

#### 2.2.1. Types of Studies

Studies with a quantitative or mixed-methods methodology of any design other than case reports were eligible for inclusion in the review. Only articles published in the English language and in a peer reviewed journal were eligible for inclusion.

#### 2.2.2. Types of Participants

We included studies examining children and adolescents (aged up to and including 17 years) with a diagnosis of FP of any aetiology. Studies investigating participants with and without FP were only eligible for inclusion if data for participants with FP was reported separately.

#### 2.2.3. Types of Intervention

Studies were included regardless of whether they were interventional or observational.

#### 2.2.4. Types of Outcome Measure

Studies were included if they reported at least one validated measure of psychosocial adjustment. Studies investigating the neurodevelopmental or neurocognitive status of participants in the absence of a measure of psychosocial adjustment were not included.

### 2.3. Information Sources

Studies were identified using electronic databases and reading the reference lists of relevant articles. The search was applied to MEDLINE (1946 onwards), CINAHL (1985 onwards), Embase (1974 onwards), PsychInfo (1806 onwards) and AMED (1985 onwards).

### 2.4. Search

**Figure 1:**
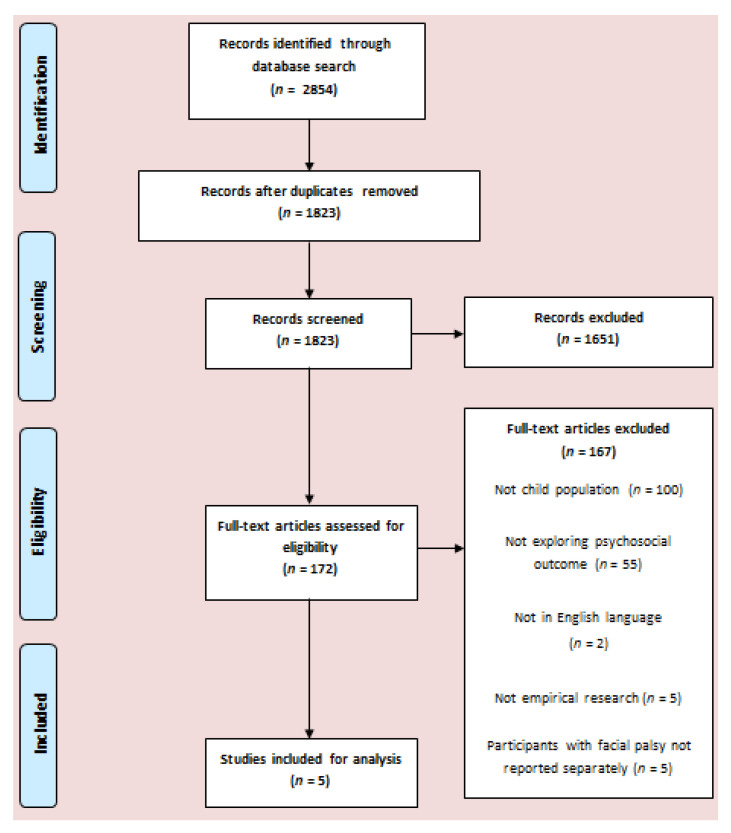
Flowchart of study selection.

We used the search terms in Table 1 to search databases. This was a replication of the search carried out in the review of Hotton et al. [12]; However eligibility criteria were adapted so that only papers exploring the paediatric, rather than adult, FP population were eligible for inclusion. The search was carried out on the 19th February 2019. An example of a full search strategy for use in Embase can be found in Figure 1.

### 2.5. Study Selection

Two reviewers developed screening questions based on the inclusion criteria, which were used to screen titles, abstracts and the full-text of papers in an independent and standardised way. Any disagreements were resolved through discussion.

### 2.6. Data Collection Process

The authors followed the preferred reporting items for systematic reviews and meta-analyses (PRISMA) guidelines in designing and reporting of studies [13]. 

### 2.7. Data Items

A data extraction form was developed to extract information from each study about: (1) psychosocial adjustment; (2) participant characteristics (age, gender and aetiology of FP); (3) outcome measures used and (4) sample size and study design.

### 2.8. Quality Assessment

The National Institutes of Health (NIH) quality assessment tool for observational cohort and cross-sectional studies [14] was used to assess the quality of the include studies. The tool was used to rate each study on a scale of 0–14, with a higher score indicating stronger scientific evidence.

## 3. Results

### 3.1. Study Selection

The database search produced 2854 papers, of which 1031 were duplicates. Of the remaining 1823 papers, 1651 papers were discarded due to not meeting inclusion criteria following the screening of titles and abstracts. Agreement between the two reviewers at the abstract screening stage was good (Kappa = 0.83). Comprehensive screening of the full text of the remaining 172 papers resulted in 5 studies meeting inclusion criteria. The phases of selection are shown in Figure 1.

### 3.2. Study Characteristics

All five studies were carried out in Germany and all recruited participants from the German Moebius Syndrome Foundation and all studies exclusively investigated the psychosocial adjustment of children and/or adolescents with Moebius syndrome. Sample sizes were generally small, ranging between 13 [15] and 31 [16]. All but one study [17] were carried out by the same research group. The mean ages ranged from 3.83 [15] to 15.20 years [18]. All studies included a majority of children with bilateral, rather than unilateral, FP. Six different measures of child psychosocial adjustment were used across the five studies and are summarised in Table 2. The most commonly used measure (*n* = 3) was the child behaviour checklist (CBCL) [19], with the German version of the strengths and difficulties questionnaire (SDQ-Deu) [20] being used in two studies. The characteristics of included studies are summarised in Table 3.

### 3.3. Risk of Bias

Table 4 shows the results from the risk of bias assessment. The included studies had a mean score of 6/14, with scores ranging between 5 and 8. All studies clearly defined their population, provided a clear research objective and uniformly applied inclusion criteria. Briegel (2012) [21] did not report their participation rate, while 3/5 did not provide justification for their sample size. No study provided an objective measure of the severity of FP symptomology.

### 3.4. Behaviour

Briegel (2007) [15] carried out the first investigation into the psychosocial outcomes of children with Moebius syndrome in a sample of children aged between 2 and 5 years (*M* = 3.83 years). Parent reports on the CBCL indicated that only 16.7% (*n* = 2) of children scored above the clinical cut-off on at least one subscale.

Briegel et al. (2010) [16] also used parent reports on the CBCL as a measure of child behaviour in an older sample of children with Moebius syndrome (median = 10.81 years). They observed higher rates of children scoring above the clinical cut-off on at least one subscale, when compared to the study by Briegel ([15]; 16.7% vs. 32.2% of the sample) and higher rates of children scoring above the clinical cut-off for total difficulties (9.7%), compared to the general population (8%).

Unfortunately, Briegel (2007) [15] and Briegel et al. (2010) [16] did not compare whether differences in CBCL subscale scores significantly differed between children with Moebius syndrome and population norms. This issue was overcome by Briegel et al. (2019) [18] who carried up a four-year follow-up study of 20 out of the 31 participants in the study by Briegel et al. (2010) [16]. They found that individuals with Moebius syndrome had significantly higher scores (more problems) on all scales of the CBCL compared to general population norms at baseline and follow-up, with the exception of thought problems at baseline and externalising problems at baseline and follow-up. A higher proportion of children with Moebius syndrome scored above the clinical cut-off on at least one subscale (55%) at follow-up compared to the baseline (32.2%), however this difference was not statistically significant. Indeed, there was no significant difference on any CBCL subscale between baseline and follow-up.

Regarding gender, Briegel [15] found that boys (*n* = 7) scored higher than girls (*n* = 6) on the aggressive behaviour, oppositional defiant problems, anxiety problems and total problems CBCL subscales, indicating greater difficulties. Briegel et al. (2010) [16] and Briegel et al. (2019) [18] did not observe any gender differences on any CBCL subscale scores. 

Only one of the three studies to use the CBCL assessed the impact of FP laterality on behaviour, with Briegel (2007) [15] observing no significant difference on any subscale between those children with bilateral FP (*n* = 9) and those with unilateral FP (*n* = 3).

The SDQ-Deu was an alternative measure of psychosocial adjustment used in two studies [17,21]. Briegel (2012) [21] found that 9/17 parents of children with Moebius syndrome aged between 9 and 15 years (*M* = 11.59 years) scored their child as being in the abnormal range on at least the SDQ-Deu subscale, with parents reporting significantly higher total difficulties compared to German population norms. However, there were no significant differences on the hyperactivity/inattention, prosocial, conduct or emotional problems subscales. Briegel (2012) [21] did not observe any statistically significant gender differences on any SDQ-Deu subscale.

Strobel and Renner (2016) [17] additionally collected self-report SDQ-Deu data in a sample with a mean age of 11.30 years, finding that self-ratings of total difficulties were significantly lower than normative data, with 20% classified as abnormal. However, self-reported total difficulties were significantly lower than parent-report ratings.

Briegel et al. (2010) [16] found that parental stress, as measured by the social orientation of parents with handicapped children (SOEBEK) [26], was positively associated with parental-reported anxious/depressed behaviour, aggressive behaviour, externalising problems and total CBCL scores. Furthermore, Briegel et al. (2019) [18] found parental strain (a broad concept including parents’ ability to handle stress), associated with greater Internalising problems and total problems on the CBCL.

### 3.5. Social Difficulties

Briegel et al. (2010) [16] compared scores on the CBCL social problems subscale, across two age groups: 4–11 years and 12–17 years. A higher percentage of parents of children aged 12–17 years reported their children to be experiencing clinically significant social problems (25%) compared to general population norms (2%), and children with Moebius syndrome aged 4–11 years (5.3%). However, the statistical significance of differences on the social problem subscale between different age groups and compared to the general population was not assessed.

Using the SDQ-Deu, Briegel (2012) [21] found five children were rated by their parents as being in the abnormal range for peer problems, a rate nearly three times higher than a normative sample, although, as with Briegel et al. (2010) [16], the statistical significance of this difference was not tested. Strobel and Renner (2016) [17] found that parents of children with Moebius syndrome reported significantly higher scores on the SDQ-Deu peer problems subscale, indicating more problems, when compared to German population norms. Self-ratings of peer problems were significantly lower than parent-report ratings.

Briegel (2012) [21] found that parent-reported peer problems significantly increased with age. However unlike Briegel et al. (2010) [16] and Briegel (2012) [21], Strobel and Renner (2016) [17] did not find any significant associations between peer problems and age and Briegel et al. (2019) [18] did not observe any significant changes on the CBCL social problems subscale at a four-year follow-up, compared to the baseline. 

### 3.6. Quality of Life

Strobel and Renner (2016) [17] used the questionnaire for measuring health-related quality of life in children and adolescents (KINDL) [24] to find that parents reported their children to have significantly poorer quality of life (QoL) in the domain of friends, but not physical wellbeing, emotional wellbeing, self-esteem, family or school, compared to normative data. The authors also observed that children’s QoL related to friends and emotional wellbeing significantly decreased with age, while QoL related to physical wellbeing, family, school, as well as overall QoL, improved with age.

### 3.7. Anxiety

Briegel (2012) [21] used the anxiety questionnaire for students (AFS; in German Angstfragebogen für Schüler) [22] to find that children with Moebius syndrome self-reported significantly lower test anxiety (anxiety related to perceived insufficiency and failure) and manifest anxiety (general anxiety symptoms) than general population norms, while there were no significant differences in anxiety related to dislike of school or social desirability.

### 3.8. Depression

Briegel (2012) [21] found children with Moebius syndrome reported significantly lower levels of depression than the general population, as measured by the depression inventory for children and adolescents (DIKJ; in German Depressionsinventar für Kinder und Jugendliche) [23].

### 3.9. Personality

Briegel (2012) [21] found that children with Moebius syndrome reported significantly lower self-perception of impulsivity, when compared to a normative sample, as measured by the self-perception dimension of the personality questionnaire for children between 9 and 14 years (PFK 9–14; in German Persönlichkeitsfragebogen für Kinder und Jugendliche) [25]. They observed no significant differences on the other subscales (subscales shown in Table 2).

## 4. Discussion

This review set out to identify the psychosocial adjustment of children and adolescents with FP. Five studies were eligible for inclusion, of which all exclusively included participants with FP due to Moebius syndrome, a form of congenital FP. Included studies measured a wide range of psychosocial constructs, including social function, behavioural difficulties, anxiety, depression and quality of life. Social difficulties were frequently reported for children with Moebius syndrome, while findings in the other areas of psychosocial adjustment were more varied.

Parents of children with Moebius syndrome were three times more likely than parents in a normative study to rate their child as having peer problems, as measured by the SDQ-Deu [21]. Similarly, Strobel and Renner (2016) [17] and Briegel et al. (2019) [18] both found parents reported their children to have greater social problems than general population norms, with Strobel and Renner (2016) [17] also observing their participants to have lower QoL related to friendships. 

The psychosocial difficulties experienced by people with Moebius syndrome, as well as others with FP of differing aetiology, may be explained by the impact of stigma on the formation of social relationships [27]. Differences in facial appearance can directly lead to stigmatisation [9]. Furthermore, difficulties producing facial expression of emotion can lead people to misinterpret the emotional state of those with FP, leading to fewer positive social interactions [8]. Facial mimicry helps people to understand the expressions of others [28] and therefore impairment in expression affects an individual’s ability to provide important social cues or influence the emotion states of others. Furthermore, FP can sometimes affect the articulation of certain speech sounds, leading to further difficulties with communication [29].

Historically researchers have argued for increased prevalence of autism spectrum disorder (ASD) in children with Moebius syndrome [30]. While this may provide an alternative explanation for the social difficulties experienced by children in the included studies, the association between ASD and Moebius syndrome has since been shown to be far less common than previously reported and previous research has been criticised for lacking representative samples and using unreliable assessment tools [31]. Furthermore, none of the participants in the included studies were identified as having a diagnosis of ASD. However, participants were typically recruited from the German Moebius Syndrome Foundation and children with severe ASD may be less likely to be active members of the foundation, potentially introducing a degree of ascertainment bias.

Despite providing initial evidence for children with Moebius syndrome experiencing greater social difficulties than the general population, it is important to consider several limitations to the discussed findings. All studies had small samples and did not include gender- or age-matched controls. Additionally, only Strobel and Renner (2016) [17] and Briegel et al. (2019) [18] assessed the statistical significance of differences with the general population. Finally, only children with Moebius syndrome were included in the studies eligible for review. Further research including children with other conditions associated with FP is required before wider conclusions can be drawn about the impact of FP on social function.

Many of the included studies found social difficulties to increase with age. For example, Briegel et al. (2010) [16] observed a higher proportion of older children experiencing social problems as measured by the CBCL, compared to younger children. Two further studies found significant associations between social factors and age, with Briegel (2012) [21] finding more peer problems with increasing age and Strobel and Renner (2016) [17] finding lower QoL related to friendships as children grew older. However, Strobel and Renner (2016) [17] did not observe a significant association between age and peer problems, while Briegel et al. (2019) [18] found no significant change in levels of social problems over time.

The potential link between age and social difficulties may be explained by the physical, social and psychological changes that occur during adolescence, which can result in an increased focus on appearance and heightened vulnerability to appearance-related concerns, potentially leading to greater social withdrawal [27]. Research with children from the general population indicates greater appearance-related social pressure with age [32]. Indeed, the development of identity [33] and identification of peer groups become increasingly important throughout adolescence [34].

Given the discrepancies across studies, the small sample sizes and absence of relevant statistical analysis in several studies, it is not possible to draw firm conclusions about the role of age in social difficulties for children with Moebius syndrome. Furthermore, participants across all studies were recruited from the same charity, meaning they may have benefitted from charity support. Finally, without the inclusion of those with FP of other aetiologies it is not possible to draw conclusions about the wider role of age in the social difficulties potentially experienced by children and adolescents with FP.

Further to the social difficulties observed across studies, Strobel and Renner (2016) [17] found parents of children with Moebius syndrome to report greater total difficulties on the SDQ-Deu compared to parents of children in the general population, while Briegel et al. (2019) [18] also found parents of older children to report that their children had greater difficulties than general population norms on most domains of the CBCL. A higher proportion of parents in the study of Briegel et al. (2010) [16] reported total problems in the clinical range on the CBCL than population norms; however no test of statistical significance was carried out.

Despite the reviewed studies indicating difficulties with social function and greater overall difficulties compared to the general population, the results of this review also highlight how many children and adolescents do not have difficulties in other psychosocial domains. Indeed, with the exception of the friends domain of the KINDL [17], all studies found the majority of children to be scoring in the non-clinical range, across all measures of behaviour or psychosocial adjustment. Furthermore, Briegel (2012) [21] found children with Moebius syndrome to have comparable levels of anxiety and depression to the general population.

The findings of this review match qualitative research that has demonstrated how although most adolescents with Moebius syndrome report having experienced bullying and social exclusion, many also report highly valued and protective social support from peers [11]. Bogart (2015) [11] found resilience to be characterised by not taking others’ responses personally and viewing Moebius syndrome as a source of strength, with some attempting to replace others’ stereotyped misconceptions with a confident personality. Other sources of resilience identified with adults with Moebius syndrome included: family support, faith, humour, sense of self, special skills, determination and networking [35]. Furthermore, it has been argued that early onset of FP, such as is the case for those with Moebius syndrome, provides adaptive advantage due children learning to use compensatory strategies, such as gesture, humour and tone of voice, in order to manage their lack of facial expression [36,37]. It is however important to note that in the absence of samples of children with FP of other aetiologies it is not possible to draw conclusions about the role of the time of onset in determining the psychosocial strengths and difficulties experienced by those with FP.

Studies also investigated the impact of gender on psychosocial adjustment. Boys scored higher than girls on the aggression, oppositional-defiant and anxiety domains of the CBCL in the youngest sample in this review [15]; however such differences were not observed in older samples [16,18,21]. This is perhaps reflective of the general population, for whom gender differences in levels of aggression have also been shown to decrease with age [38]. Again, it is important to note that the samples in the included studies were small, potentially leaving them under-powered to observe statistically significant effects.

Briegel et al. (2007) [15] carried out the only study to compare children with bilateral FP to those with unilateral FP, finding no significant differences on behavioural outcomes. This is consistent with findings from the wider adult FP literature, which also indicate that the objective severity of symptoms does not necessarily predict psychosocial adjustment (see [12], for a review). However, most children with Moebius syndrome have bilateral FP, whereas the majority of the wider paediatric FP population have unilateral FP [39]. Further studies of FP of other aetiologies would be useful in examining whether the lack of association between objective severity of FP and psychosocial adjustment is consistent, despite the cause.

Two studies [16,17,18] highlighted the significant relationship between parental self-reported stress and increased parental-reporting of child behavioural difficulties. Although neither study observed a significant difference in the levels of stress between parents of children with FP and parents of children in the general population, it is important to consider experiences of parents caring for a child with medical needs. Research has shown that elevated parental stress can have a negative impact on children’s socioemotional development, and subsequently their behaviour [40]. Indeed, Guajardo et al. (2009) [40] argued that parents with lower levels of stress may be more able to engage their children in parent–child interactions, and subsequently support their socioemotional development, something that may be of increased importance for children with impaired facial expression of emotion, such as those with FP. Furthermore, child behaviour problems have been shown to lead to parental stress [41], highlighting how the relationship between child behaviour and parental stress may be bidirectional in nature.

### 4.1. Clinical Implications 

This review highlights the importance of routinely screening children with Moebius syndrome for psychosocial difficulties, particularly with regards to social problems. Those identified as having difficulties may benefit from psychological intervention. Michael et al. (2015) [42] conducted a pilot social skills intervention with five adolescents with Moebius syndrome. It included a group-based discussion of challenges, education about building confidence, navigating job interviews and relationships and training on alternative expression. There was preliminary evidence for increases in participant expressive behaviour, interaction partners’ expressive behaviour and observer-rated rapport.

Other evidence for the positive impact of psychological interventions in the wider FP population comes from Hotton et al. (2019) [43], who highlighted the benefits of a single-session parent and child workshop for children with FP of any aetiology, particularly with regards to parents’ confidence in talking to their children about their facial difference. 

As well as indicating the importance of screening and psychological intervention, this review highlighted the role that parental factors may play in their children’s psychosocial adjustment. It is therefore recommended that Clinical Psychologists with specialist knowledge of Moebius syndrome, and other forms of FP, work within specialist FP services, in order to identify and provide psychological treatment for those children, adolescents and their families experiencing psychosocial difficulties.

### 4.2. Limitations and Future Directions

Despite this review being the first of its kind to evaluate the psychosocial adjustment of children and adolescents with FP, only studies investigating the impact of Moebius syndrome met the inclusion criteria. This is not representative of the wider paediatric FP population, as FP due to causes such as Bell’s palsy, trauma and hemifacial macrosomia, amongst others, are more common than Moebius syndrome [1]. Furthermore, Moebius syndrome is typically associated with many other physical difficulties, in addition to FP (Table 3). Therefore psychosocial difficulties reported in the included studies could be due to confounding factors other than FP. 

The absence of any studies investigating the impact of FP of other aetiologies on psychosocial adjustment during childhood limits the ability to draw wider conclusions. Future research into the psychosocial adjustment of children and adolescents with FP and no other known comorbidities, as well as direct comparison between those with congenital and acquired FP, is required to understand the specific impact of FP on children and adolescents’ psychosocial functioning.

A further limitation of the current review was that it did not include studies investigating populations, including those with genetic conditions, where some, but not all, of the individuals had a diagnosis of FP (unless those with FP were investigated separately). An example of such a population is children and adolescents with CHARGE syndrome, of whom an estimated 50–90% have FP [44]. Although the psychosocial difficulties experienced by many of the children and adolescents with CHARGE syndrome are well documented [45], no published research identified in the current review separately investigated those with CHARGE syndrome and FP. It would therefore be beneficial for future research into the psychosocial adjustment of children and adolescents with conditions often associated with FP, such as CHARGE syndrome, to directly compare the outcomes of those with and without FP. This will further help improve the understanding of the specific impact of FP, compared to the impact of other comorbidities.

Four out of the five included studies came from the same research group [15,16,18,21] and all recruited from the German Moebius Syndrome Foundation. This significantly limits heterogeneity with regards to the participants being investigated and introduces a large degree of selection bias. In addition, cultural norms may affect psychosocial adjustment to FP and given that all participants were German, it is not possible to confirm whether the findings of this study generalize to people from other cultures. Furthermore, all studies had small samples, with no studies carrying out a prospective power analysis.

The outcome measures used across studies were often in the German language, which limits the ability to replicate findings across other studies in other countries. There was a degree of inconsistency with regards to the measures used, and none were designed specifically for individuals with FP, so may lack the sensitivity to detect condition-specific issues. Future research would therefore benefit from the development of measures specific to FP and the consistent application of measures across studies.

Only one study had a longitudinal design [18] and no studies included a (matched) control group. These limitations affect our ability to draw firm conclusions about how psychosocial difficulties change over time and how they differ from the general population, and should be addressed in future research.

All of the included studies used a parental report as a measure of psychosocial adjustment, while Briegel (2012) and Strobel and Renner (2016) [17] also included self-report measures. Research from other areas of visible difference has shown how parental reports of psychosocial adjustment do not necessarily match self-reports, with parents often over-reporting difficulties [46]. Further, self-reports have also been shown to differ from observational evidence [47], highlighting the importance of future research using parental report in conjunction with other sources of information, including self-report, teacher-report, as well as more objective assessments.

Future research would therefore benefit from several considerations. Firstly, research should be longitudinal in nature, providing the opportunity to observe how adjustment changes over time. Secondly, studies should include children with FP of varying aetiologies and be sufficiently powered to allow for comparison between different aetiologies. Thirdly, research should control for any additional physical or cognitive difficulties, as well as to report on the objective severity of FP symptoms. Fourthly, participants should be recruited from a variety of countries, in order to better explore the impact of cultural norms. Fifthly, research should include a combination of self- and parent-reported outcome measures, as well as ideally a combination of generic measures of psychosocial adjustment and those designed specifically for children with FP.

Finally, there is a need for the development and evaluation of further evidence-based psychosocial interventions, in order to help children and adolescents and their parents to adjust to the functional and appearance aspects of conditions such as Moebius syndrome.

## 5. Conclusions

FP is a condition that has wide-ranging functional and psychological consequences, however, to date the psychosocial impact of the condition in childhood and adolescence has only been investigated in those with Moebius syndrome. Studies provide some indication of specific difficulties with social functioning, which may increase with age. Further research with children with FP of varied aetiologies using a combination of outcome measures is required. These measures should include both facial-palsy specific measures and also generic measures to permit comparison with the general population; this combined approach will enable comprehensive assessment of the specific psychosocial impact of the condition.

## Figures and Tables

**Table 1:** Search terms used in the literature search.

Subheading	Search Terms
**Population**	Facial palsy-facial paralys *, facial pals *, facial pares *, hemi-facial paralys *, hemi-facial pals *, hemi-facial pares *, Bell * Pals *, Ramsay Hunt, Mo?bius
**Outcome**	Psychosocial- psychology, psychiatry, psych *, mental disorders, anxiety, anxious, depress *, distress *, mood, emotion *, confidence, self-concept, self-perception, self-esteem, self-image, self-worth, body image, appearance

*Note:* * indicates truncation.

**Table 2:** Child outcome measures used in included studies.

Outcome Measure	Construct Measured	Subscales	Parent-or Self-Report
AFS	Anxiety	Test Anxiety Manifest Anxiety Dislike of School Social Desirability	Self-report
CBCL	Behaviour	Social Withdrawal Somatic Complaints Anxiety/Depression Social Problems Thought Problems Attention Problems Delinquent Behaviour Aggressive Behaviour	Parent-report
DIKJ	Depression	N/A	Self-report
KINDL	Quality of Life	Physical Wellbeing Emotional Wellbeing Self-Esteem Family Friends School	Parent- and self-report
PFK 9–14	Personality	General Anxiety Belief in Own Opinions Decisions and Plans Self-Perception of Impulsivity Tendency to Overestimate One’s own Capabilities Feeling of Inferiority	Self-report
SDQ-Deu	Psychosocial Adjustment	Emotional Problems Conduct Problems Hyperactivity/Inattention Peer Problems Prosocial Behaviour	Parent-and self-report

Note: AFS—Angstfragebogen für Schüler [22]; CBCL—Child Behaviour Checklist [19]; DIKJ—in German Depressionsinventar für Kinder und Jugendliche [23]; KINDL—Questionnaire for Measuring Health-Related Quality of Life in Children and Adolescents [24]; PFK—Persönlichkeitsfragebogen für Kinder und Jugendliche [25]; SDQ-Deu—German version of the Strengths and Difficulties Questionnaire [20].

**Table 3:** Characteristics of included studies.

Authors	Country	Sample Size n (n Female)	Age in Years *M* (SD)	Diagnosis	Comorbidities	Design and Setting	Measures	Main Finding(s)
Briegel, Hofmann, and Schwab (2007)	Germany	13 (6)	3.83 (Range = 2.08– 5.92)	Moebius syndrome: bilateral facial paralysis (*n* =9) unilateral facial paralysis (*n* = 3)	strabismus (*n* = 8) ptosis (*n* = 4) malformation of hands (*n* = 7) malformation of feet (*n* = 5)	Cross-sectional; recruitment from the German Moebius Foundation	CBCL (parent-report)	16.7% (*n* = 2) scored in clinical range on at least one scale Boys scored higher (more problems) than girls on: Aggressive Behaviour, Total Problems, Oppositional Defiant Problems and Anxiety Problems
Briegel, Hofmann, and Schwab (2010)	Germany	31 (19)	Median = 10.58 (Range = 4.58 – 17.00)	Moebius syndrome: bilateral facial paralysis (*n* =22) unilateral facial paralysis (*n* = 9)	strabismus (*n* = 19) malformation of feet (*n* = 15) malformation of hands (*n* = 11) hearing loss (*n* = 7) Poland sequence (*n* = 4) ptosis (*n* = 3) congenital heart defect (*n* = 3) arthrogryposis (*n* = 3) scoliosis (*n* = 2) Pierre-Robin sequence (*n* =2) seizures (*n* = 1)	Cross-sectional; recruitment from the German Moebius Foundation	CBCL (parent-report)	32.2% (*n* = 10) scored in clinical range on at least one scale No significant differences between genders on any scale Social Problems more common among older children
Briegel (2012)	Germany	17 (8)	11.59 (1.87)	Moebius syndrome: bilateral facial paralysis (*n* = 15) unilateral facial paralysis (*n* = 2)	bilateral impairment of ocular abduction (*n* = 16) bilateral impairment of ocular adduction (*n* = 6) congenital bilateral hypoglossal paralysis (*n* = 14) malformations of feet (*n* = 9) malformation of hands (*n* = 5) ptosis (*n* = 3) congenital heart defect (*n* = 3) Poland syndrome (*n* = 3) hearing impairment (*n* = 2) Kallmann’s syndrome (*n* = 1)	Cross-sectional; recruitment from the German Moebius Foundation	AFS	Lower Test Anxiety and Manifest Anxiety than normative data
							DIKJ	Lower Total Depression than normative data
							SDQ- Deu	52.9% (*n* = 9) scored in the abnormal range on at least one scale Social Problems significantly increased with age
Strobel and Renner (2016)	Germany	26 (11)	11.30 (4.50)	Moebius syndrome: bilateral facial paralysis (*n* = 19) unilateral facial paralysis (*n* = 7)	abducens paralysis (*n* = 26) no other comorbidities reported	Cross-sectional; recruitment from the German Moebius Syndrome Foundation	KINDL	Significantly greater difficulties with Friends compared to normative data (parent- and self-report) Significantly greater difficulties with Friends and Emotional Well-Being with increased age
							SDQ- |Deu	Significantly higher Total Difficulties (parent-report) and Peer Problems (parent- and self-report) than normative data
Briegel, Heinzel-Guttenbrunner, and Beate (2019)	Germany	26 (14)	15.20 (3.48)	Moebius syndrome: bilateral facial paralysis (*n* = 17) unilateral facial paralysis (*n* = 9)	bilateral abducens paralysis (*n* = 25) unilateral abducens paralysis (*n* = 1) malformation of feet (*n* = 13) malformation of hands (*n* = 10) strabismus (*n* = 8) Poland sequence (*n* = 5) arthrogryposis (*n* = 4) hearing loss (*n* = 3) congenital heart defect (*n* = 3) scoliosis (*n* = 3) seizures (*n* = 2) ptosis (*n* = 1) Pierre-Robin sequence (*n* = 1)	Cohort; four-year follow-up to participants in study described by Briegel et al. (2010); German Moebius Syndrome Foundation	CBCL (parent report)	Significantly higher scores (more problems) than the general population on all scales apart from Externalising problems 55% (*n* = 11) scored in clinical range on at least one scale No significant changes between scores at T1 (Briegel et al. 2010) and T2 (Briegel et al. 2019)

**Table 4:** Risk of bias for included studies (NIH quality assessment tool for observational cohort and cross-sectional studies; National Institutes of Health, 2014).

	Study				
	Briegel et al. (2007) [15]	Briegel et al. (2010) [16]	Briegel (2012) [21]	Strobel and Renner (2016) [17]	Briegel et al. (2019) [18]
1. Was the research question or objective in this paper clearly stated?	Yes	Yes	Yes	Yes	Yes
2. Was the study population clearly specified and defined?	Yes	Yes	Yes	Yes	Yes
3. Was the participation rate of eligible persons at least 50%?	Yes	Yes	NR	Yes	Yes
4. Were all the subjects selected or recruited from the same or similar populations (including the same time period)? Were inclusion and exclusion criteria for being in the study prespecified and applied uniformly to all participants?	Yes	Yes	Yes	Yes	Yes
5. Was a sample size justification, power description, or variance and effect estimates provided?	Yes	No	No	Yes	No
6. For the analyses in this paper, were the exposure(s) of interest measured prior to the outcome(s) being measured?	No	No	No	No	No
7. Was the timeframe sufficient so that one could reasonably expect to see an association between exposure and outcome if it existed?	Yes	Yes	Yes	Yes	Yes
8. For exposures that can vary in amount or level, did the study examine different levels of the exposure as related to the outcome (e.g., categories of exposure, or exposure measured as continuous variable)?	No	No	No	No	No
9. Were the exposure measures (independent variables) clearly defined, valid, reliable, and implemented consistently across all study participants?	Yes	Yes	Yes	Yes	Yes
10. Was the exposure(s) assessed more than once over time?	No	No	No	No	Yes
11. Were the outcome measures (dependent variables) clearly defined, valid, reliable, and implemented consistently across all study participants?	NR	NR	NR	Yes	NR
12. Were the outcome assessors blinded to the exposure status of participants?	NR	NR	NR	NR	NR
13. Was loss to follow-up after baseline 20% or less?	NA	NA	NA	NA	Yes
14. Were key potential confounding variables measured and adjusted statistically for their impact on the relationship between exposure(s) and outcome(s)?	No	No	No	No	No
Overall Score (0–14)	7	6	5	8	8

Note: NR = Not Reported, NA = Not Applicable.
